# Gestational Age, Birth Weight, and Neurocognitive Development in Adolescents in Tanzania

**DOI:** 10.1016/j.jpeds.2021.04.036

**Published:** 2021-04-24

**Authors:** Nandita Perumal, Karim P. Manji, Anne Marie Darling, Rodrick R. Kisenge, Ingrid Kvestad, Mari Hysing, David C. Belinger, Willy Urassa, Tor A. Strand, Christopher P. Duggan, Wafaie W. Fawzi, Christopher R. Sudfeld

**Affiliations:** 1 Department of Global Health and Population, Harvard T.H. Chan School of Public Health, Boston, MA; 2 Department of Pediatrics and Child Health, Muhimbili University of Health and Allied Sciences, Dar es Salaam, Tanzania; 3 Regional Center for Child and Youth Mental Health and Child Welfare, NORCE Norwegian Research Center; 4 Department of Psychological Science, Faculty of Psychology, University of Bergen, Bergen, Norway; 5 Department of Neurology, Boston Children’s Hospital, Boston, MA; 6 Department of Microbiology and Immunology, Muhimbili University of Health and Allied Sciences, Dar es Salaam, Tanzania; 7 Department of Research, Innlandet Hospital Trust, Lillehammer, Norway; 8 Faculty of Medicine, Centre for International Health, University of Bergen, Bergen, Norway; 9 Division of Gastroenterology, Hepatology and Nutrition, Boston Children’s Hospital and Harvard Medical School; 10 Department of Nutrition, Harvard T.H. Chan School of Public Health; 11 Department of Epidemiology, Harvard T.H. Chan School of Public Health, Boston, MA

## Abstract

**Objectives:**

To investigate the association between gestational age, birthweight, and birthweight adjusted for gestational age, with domains of neurocognitive development and behavioral problems in adolescents in Tanzania.

**Study design:**

Data from a long-term follow-up of adolescents aged 11-15 years born to women previously enrolled in a randomized controlled trial of prenatal multiple micronutrient supplementation in Dar es Salaam, Tanzania, were used. A battery of neurodevelopmental tests were administered to measure adolescent general intelligence, executive function, and behavioral problems. The INTERGROWTH-21^st^ newborn anthropometric standards were used to derive birthweight for gestational age z-scores. We assessed the shape of relationships using restricted cubic splines and estimated the associations of gestational age, birthweight, and birthweight for gestational age z-score with adolescent development using multivariable linear regressions.

**Results:**

Among adolescents studied (n = 421), higher gestational age (per week), birthweight (per 100 grams), and birthweight for gestational age z-score (per SD) were linearly associated with higher intelligence score (adjusted standardized mean difference, 0.05 SD [95% CI, 0.01-0.09], 0.04 SD [95% CI, 0.02-0.06], and 0.09 SD [95% CI, 0.01-0.17], respectively). Birthweight and birthweight for gestational age z-score, but not gestational age, were also associated with improved executive function. Low birthweight (<2500 g) was associated with lower intelligence and executive function scores. Associations between birthweight and executive function were stronger among adolescents born to women with higher education.

**Conclusions:**

The duration of gestation and birthweight were positively associated with adolescent neurodevelopment in Tanzania. These findings suggest that interventions to improve birth outcomes may also benefit adolescent cognitive function.

Globally, approximately 14 million (10.6%) live births are estimated to be preterm (birth <37 weeks gestational age), 20 million (14.6%) are estimated to be low birthweight (<2500 g at birth), and 23 million (19.3%) are estimated to be small for gestational age (birthweight for gestational age <10th of the standard reference population).^1–3^ Countries in sub-Saharan Africa and South Asia bear a disproportionate burden of these perinatal adversities.^1–3^ Children who are born too soon or too small are at a greater risk of mortality, poor growth, and suboptimal neurodevelopment in early childhood as well as lower academic performance later in life.^3–15^ Cumulatively, these deficits may translate into reductions in educational attainment and economic gains across the life course for individuals and populations.^16^

The majority of evidence on the long-term neurodevelopmental impacts of adverse perinatal outcomes however are based on studies from high-income settings and restricted to populations of very preterm-born (ie, <32 weeks gestational age) or very low birthweight infants (<1500 g).^17^ There are few data on the relationship of adverse birth outcomes with adolescent neurocognitive development from low- and middle-income countries (LMICs) where the burden of these outcomes is greatest. In addition, although most studies evaluate the impacts on cognitive development and intelligence scores, few studies have examined the association between birth outcomes and executive function (encompassing abilities such as intentional control, cognitive flexibility, attention, and working memory) or behavioral problems among adolescents in LMICs.^18^ Higher performance in measures of executive function have been linked to improved health and developmental outcomes in later life, including academic achievement, social competence, ability to cope with stress, and physical health.^18^

In this study, we used data from a prospective birth cohort in Tanzania to investigate the association between gestational age, birthweight, and birthweight adjusted for gestational age with domains of neurodevelopment, including general intelligence, executive function, and behavioral problems, among adolescents 11-15 years of age. We further examined whether maternal education and adolescent sex modified the strength of the relationships between adverse birth outcome and adolescent development.

## Methods

### Study Population

We used secondary data from a follow-up study of adolescents born to women previously enrolled in a double-blind randomized controlled trial of daily prenatal multiple micronutrient supplementation conducted between 2001 and 2005 in Dar es Salaam, Tanzania. The trial procedures and primary findings are published in detail elsewhere.^19^ Briefly, 8428 HIV-uninfected pregnant women between 12 and 28 weeks gestation were randomized to receive either a daily multiple micronutrient supplement or placebo during pregnancy to investigate the effects on perinatal outcomes, including low birthweight, preterm birth, and fetal death. The trial findings showed that daily supplementation with multiple micronutrients during pregnancy reduced the risk of low birthweight and small for gestational age, but did not significantly reduce the incidence of preterm birth or fetal death.^19^ Subsequently, all children born to women in the trial were eligible for recruitment into the adolescent follow-up study at 11-15 years of age, which aimed to assess the long-term effects of prenatal multiple micronutrient supplement on physical growth and neurocognitive development.^20^ A detailed description of the adolescent follow-up study procedures and primary findings has been published elsewhere.^20,21^ Written informed consent was obtained from mothers or primary caregivers and assent from all adolescents enrolled in the follow-up study. Ethical approvals for the follow-up study were received from Institutional Review Boards at the Harvard T.H Chan School of Public Health, the National Institute of Medical Research and the Muhimbili University of Health and Allied Sciences in Tanzania.

### Exposure Measure

Newborn weight was measured to the nearest 10 g by trained research midwives at the time of delivery.^19^ Gestational age was measured using the date of last menstrual period recorded at enrollment into the parent prenatal multiple micronutrient supplement trial. We used standard definitions of preterm birth (<37 weeks gestational age) and low birthweight (<2500 g) to classify birth outcomes. Small for gestational age was defined as birthweight for gestational age below the 10th percentile of the sex- and gestational age-matched reference population based on the INTERGROWTH-21^st^ very preterm size at birth references and newborn size standards.^22^ Average for gestational age was defined as being between the 10th and 90th percentiles and large for gestational age defined as the 90th percentile or higher of the reference population. We further categorized adolescents into combined categories of preterm/term and birthweight for gestational age percentile categories for comparison.

### Outcome Measures

We assessed 3 domains of adolescent neurodevelopment: general intelligence, executive function, and behavioral problems. Detailed descriptions of the neurodevelopmental test battery, as well as procedures for translation to local language (Kiswahili), local adaptation and validation were published previously.^20,23^ Briefly, the East Africa Neurodevelopmental Battery was designed for use in low-resource settings to assess core constructs of cognitive ability, namely general intelligence, executive function, and literacy skills, using culturally appropriate tools, and has been adapted and validated for use in Bangladesh, Ghana, and Tanzania.^22^ Tests to assess general intelligence included, the Atlantis, Footsteps, Hand movement, Kilifi naming test, story completion, Koh’s Block Design test, and Verbal Fluency test. The literacy and numeracy test, go/no go test, people search, Rey-Osterrieth complex figure, and Shift, were used to evaluate the constructs of working memory, attention, inhibitory control, and achievement (a measure of application of skills in school-based learning).^21,23^ In addition, we used the parent-reported Strengths and Difficulties Questionnaire (SDQ) and the Behavior Rating Inventory of Executive Function (BRIEF) to assess adolescent mental health and behavioral problems covering executive function. These tests were carefully selected as they are well-validated for use across different settings and are sensitive measures of different aspects of mental and behavioral health among adolescents.^20^ Inter-rater reliability was assessed for each development test in a subgroup of 18 children by having 2 interviewers assess the same child at the same time every month during data collection.^21^ Agreement between interviewers for all tests were high (Kappa coefficient >0.60), except for the Kilifi naming test and verbal fluency tests, which had moderate reliability (kappa coefficients 0.42 and 0.47, respectively).^21^

The 3 domains of neurodevelopment were assessed by grouping domain-specific individual neurodevelopment test scores into an average composite z-score for each given domain. For example, general intelligence was assessed by converting the individual subtests scores (Atlantis, Footsteps, Hand movement, Kilifi naming test, Koh’s Block design test, Story completion, and verbal fluency) to z-scores and then averaging the scores to create a composite score for intelligence. Similar approaches were taken to generate the executive function z-score, which combined scores from the literacy and numeracy tests, go/no go test, people search, Rey-Osterrieth complex figure, and Shift, and the behavioral problems z-score, which combined the total problem score from SDQ and the BRIEF questionnaires. This analytical approach to combine subtest z-scores has been the preferred method in previous studies because it decreases the risk of type I errors owing to multiple testing.^21,24^ For intelligence and executive function, a higher score suggests a better outcome, whereas for the behavioral problems score, a higher score was indicative of poorer outcome.

### Statistical Analyses

We first examined the shape of the associations between the exposures (gestational age at birth, birthweight, and birthweight for gestational age z-score) and outcomes (general intelligence, executive function, and behavioral problem scores) at 11-15 years of age separately. Restricted cubic splines were used to assess possible nonlinearity of associations between perinatal outcomes and adolescent development.^25^ The likelihood ratio test was used to compare the model with only the linear term to the model with the linear and cubic spline terms; continuous exposure variables were categorized into quartiles if models suggested significantly nonlinear relationships.

Based on the shapes of the relationships, we used multivariable linear regression models to estimate the change in standardized mean difference (SMD) with 95% CI in each domain of adolescent neurodevelopment (general intelligence, executive function, and behavioral problems) as a function of gestational age at birth, birthweight, and birthweight for gestational age z-score, separately. Given the nonlinearity of association in the spline analysis, we used quartiles of birthweight to model the association between birthweight and behavioral problem score. Models were adjusted for common confounders of the association between birth outcomes and adolescent development based on previous literature, including adolescent age at the time of assessment, sex, maternal age, maternal education, maternal marital status, maternal parity, wealth quartile, alcohol consumption in the last month, and maternal supplementation regimen (placebo vs micronutrient supplementation). We did not adjust for any postnatal factors because such factors may be on the causal pathway as mediators between birth outcomes and adolescent development or associated with potential mediators. We used interaction terms to explore whether the relationship between perinatal outcomes and adolescent neurodevelopment was modified by maternal education or child sex. The likelihood ratio and Wald tests were used to assess the statistical significance of interaction terms. To examine the potential for selection bias owing to loss to follow-up, we compared baseline caregiver and child characteristics among adolescents who were enrolled in the follow-up study compared with those who were lost to follow-up. In sensitivity analyses, we further used inverse probability of censoring stabilized weights to account for potential selection bias owing to loss to follow-up and to assess consistency of inferences based on our primary analyses. All *P* values were 2-sided with an alpha of 0.05. Statistical analyses were conducted using SAS version 9.2 (SAS Institute) and Stata version 14 (StataCorp).

## Results

**Figure 1.**
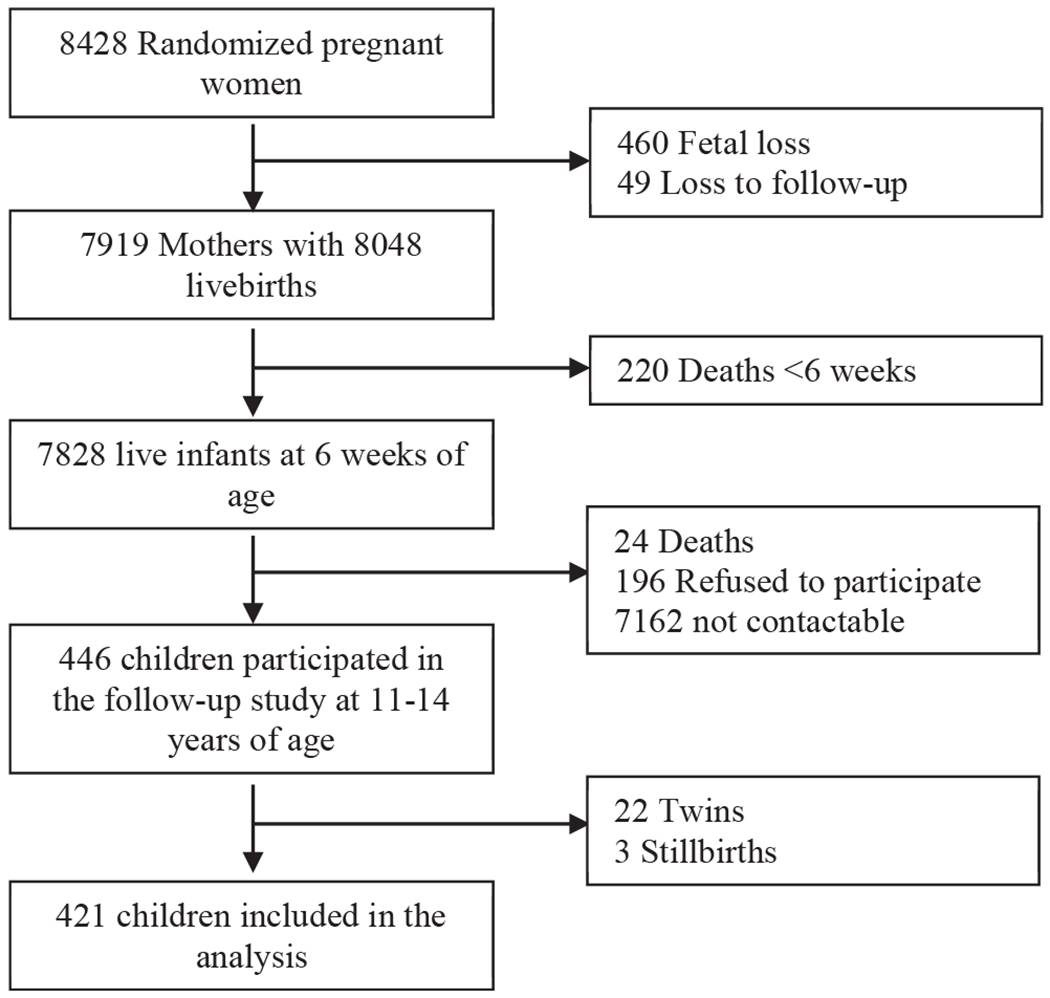
Flowchart of participants enrolled in the adolescent follow-up study and analytical sample.

Of the 8428 women enrolled in the prenatal micronutrient supplementation trial, 446 adolescents were enrolled in the follow-up study at 11-15 years of age (Figure 1; available at www.jpeds.com). The primary reason for the loss to follow-up was due to an inability to contact original trial participants at the time of the adolescent follow-up study, which occurred 10-14 years after the parent trial. Adolescents who had been singleton births, had data on gestational age or birthweight, and underwent neurodevelopmental assessment were included in the present study (n = 421). Characteristics of mothers and adolescents who participated in the follow-up study are summarized in Table I. Women were on average 28.1 ± 4.9 years old when they were recruited in pregnancy, had completed primary school (63%), were married (92%), and were multiparous (50%). Adolescents who participated in the follow-up study were on average born at 39.6 ± 2.4 weeks (range, 29-43 weeks) and had a mean birthweight of 3210 ± 498 g. In this study, the prevalence of preterm birth, low birthweight, and small for gestational age (birthweight <10th percentile) was 13.0%, 3.8%, and 17.0%, respectively. The majority of adolescents were born average for gestational age (82%). The mean age at neurodevelopmental assessment was 13.1 ± 0.9 years. Adolescents who were enrolled in the follow-up study compared with those lost to follow-up were less likely to be born preterm or low birthweight and tended to be born to women who were older, multiparous, in a higher wealth quintile, and slightly more likely to report consuming alcohol once or more per week (Table II; available at www.jpeds.com).

**Figure 2.**
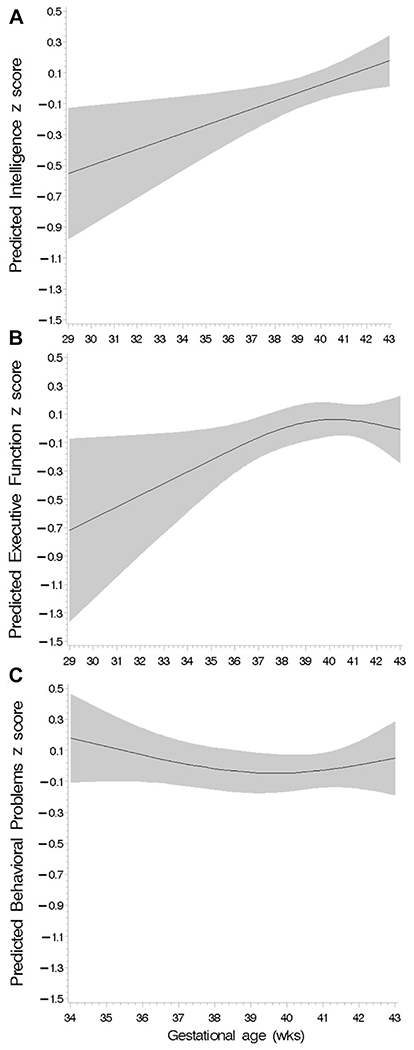
Spline analysis of the covariate-adjusted association between gestational age (weeks) and, **A,** intelligence score (*P* for linear association = .01); **B,** executive function score (*P* for linear association = .09), and **C,** behavioral problems score (*P* for linear association = .44).

**Figure 3.**
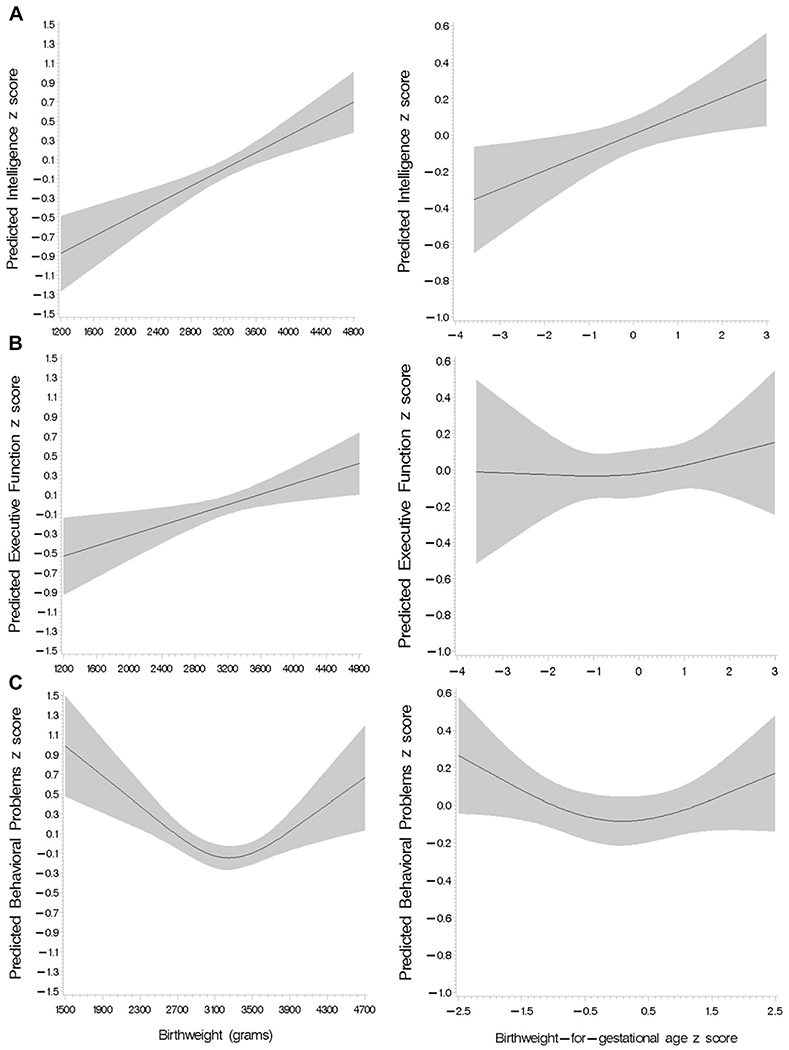
Spline analysis of the covariate-adjusted associations between continuous birthweight (grams) (left) and birthweight for gestational age (right) with **A,** intelligence score (*P* for linear association < .001 and .01, respectively), **B,** executive function score (*P* for linear association = .01 and .48, respectively), and **C,** behavioral problems score (*P* for nonlinear association = .01 and .65, respectively).

**Figure 4.**
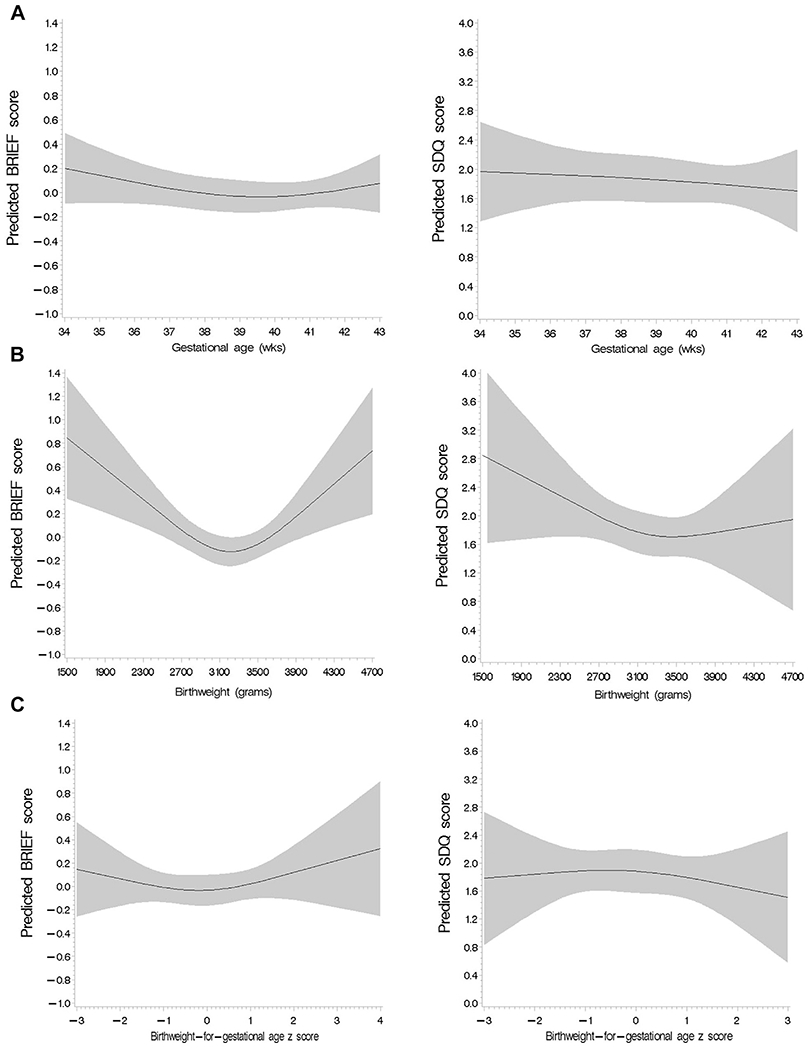
Spline analysis of the covariate-adjusted association between continuous, **A,** gestational age, **B,** birthweight (grams), and **C,** birthweight for gestational age with the BRIEF (left) and SDQ (right) scores.

We first examined the shape of the relationship between birth outcomes and adolescent neurodevelopment. Gestational age had a statistically significant linear relationship with intelligence score (*P* = .01); however, the spline analysis could not definitively establish linearity or nonlinearity of associations between gestational age and executive function and behavioral problem scores (Figure 2). Birthweight was linearly associated with intelligence and executive function domain scores, whereas the association with behavioral problems score was significantly nonlinear (Figure 3). Similarly, birthweight for gestational age was linearly associated with intelligence score, but the shape of the associations with executive function and behavioral problems scores were neither significantly linear nor nonlinear based on spline analysis (Figure 3). We further examined the shape of the relationship between birth outcomes and behavioral problems score disaggregated by SDQ and BRIEF domain scores (Figure 4; available at www.jpeds.com).

The shape of these relationships informed the multivariable models to estimate the associations between birth outcomes and neurodevelopmental domains (Table III). Adolescent intelligence score was positively associated with gestational age (adjusted SMD [aSMD], 0.05; 95% CI, 0.01-0.09 per week), birthweight (aSMD, 0.04; 95% CI, 0.02-0.06 per 100 g), and birthweight for gestational age z-scores (aSMD, 0.09; 95% CI, 0.01-0.17 per 1 SD), although the magnitude of the associations were small. Birthweight and birthweight for gestational age, but not gestational age, were also associated with improved executive function at 11-15 years of age (aSMD, 0.03; 95% CI, 0.01-0.05 per 100 g increase, and aSMD 0.08; 95% CI, −0.00 to 0.16 per 1 SD increase, respectively). The behavioral problems score at 11-15 years was not associated with gestational duration, although, adolescents who were born preterm had a higher behavioral problems score (aSMD, 0.28; 95% CI, −0.01 to 0.58) compared with adolescents born at ≥37 weeks gestation. Similarly, adolescents who were born low birthweight, compared with those born at ≥2500 g, had higher behavioral problems score (aSMD, 0.75; 95% CI, 0.24-1.27) and lower intelligence and executive function scores (Table III). Although we observed a U-shaped relationship between birthweight and behavioral problem score, confidence intervals of the associations between birthweight >3200 g, relative to reference birthweight of 2900-3200 g, crossed the null (Table III). Being born small for gestational age alone was not associated with neurodevelopmental scores at 11-15 years of age; however, adolescents who were born both preterm and small for gestational age had large deficits in executive function (aSMD, −1.10; 95% CI, −1.10- to −0.06) and higher behavioral problems score (aSMD, 1.38; 95% CI, 0.38-2.39) compared with their term-born average for gestational age counterparts (Table IV; available at www.jpeds.com); these findings, however, are based on a very small number of children. In sensitivity analyses using inverse probability stabilized weights to account for loss to follow-up, measures of associations remained similar and inferences were qualitatively unchanged (Table V; available at www.jpeds.com).

**Figure 5.**
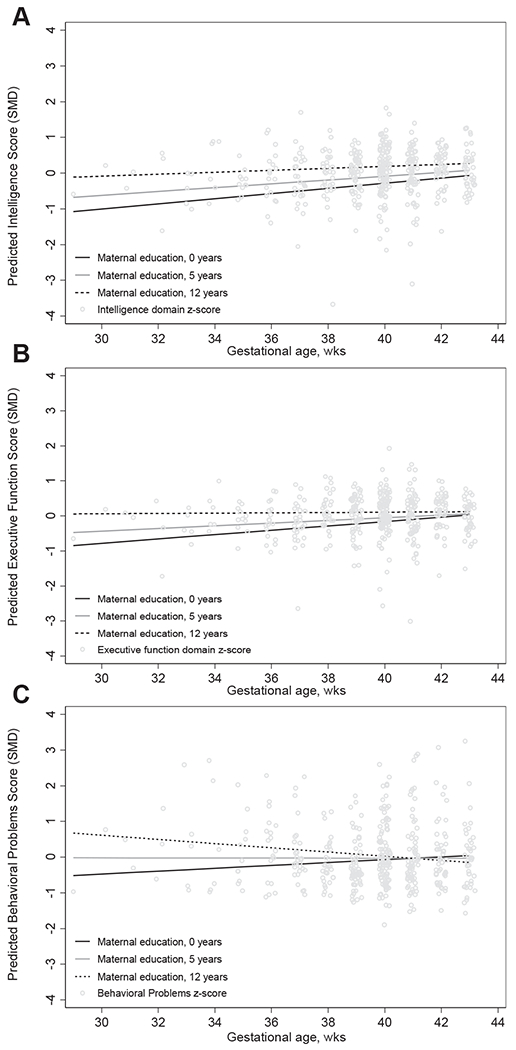
Predicted marginal effect of gestational age on intelligence score, **A;** executive function score, **B;** and behavioral problems score, **C,** among adolescents born to women with varying years of education after multivariable adjustment for adolescent age, sex, maternal age, maternal marital status, maternal parity, wealth quartile, alcohol consumption, and supplementation regimen. *P* values for continuous interaction terms between gestational age and maternal education were .55, .44, and .20 for intelligence, executive function, and behavioral problems domain scores, respectively.

**Figure 6.**
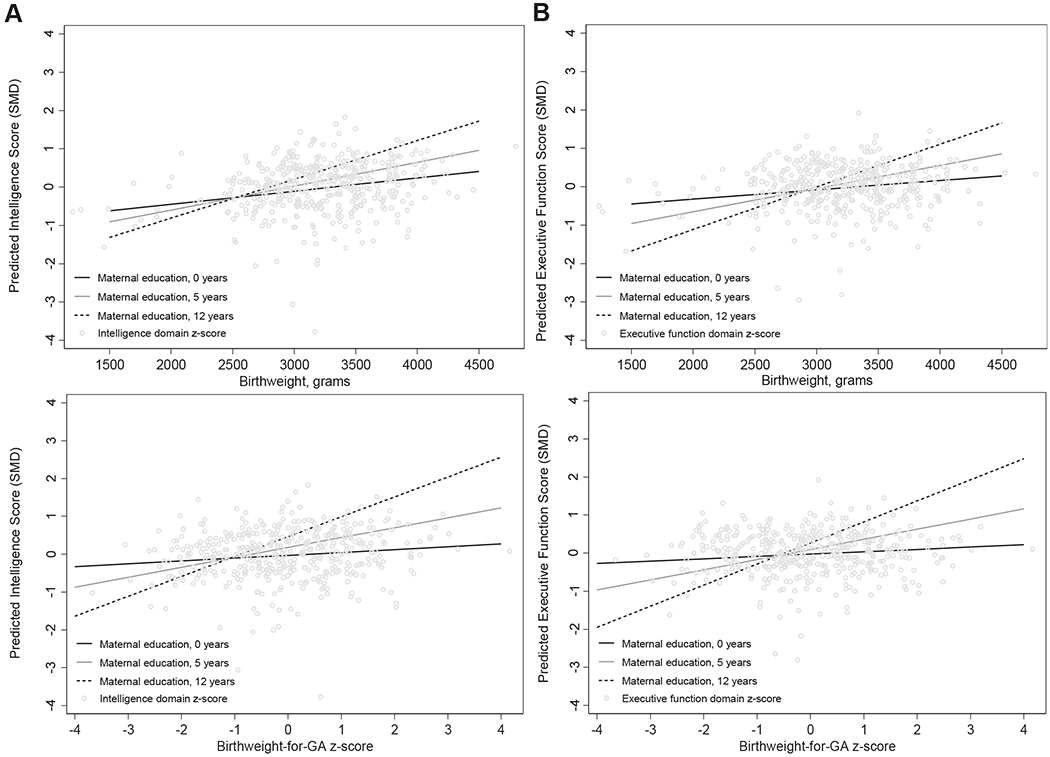
Predicted marginal effect of birthweight (row) and birthweight for gestational age (bottom) on **A,** intelligence score and **B,** executive function score among adolescents born to women with varying years of education. *P* values for continuous interaction terms between birthweight and maternal education were .07 and .02 for intelligence and executive function domain score, respectively. *P* values for continuous interaction terms between birthweight for gestational age z-score and maternal education were .01 and .002 for intelligence and executive function domain scores, respectively. Including a continuous interaction term with maternal education for any domain however did not improve model fit (based on likelihood ratio test).

Maternal education did not significantly modify the associations between gestational age and adolescent developmental scores (Table VI and Figure 5; both available at www.jpeds.com). However, associations between birthweight for gestational age and executive function and behavioral problems scores were stronger among adolescents born to women with higher levels of education (Figure 6; available at www.jpeds.com). Interestingly, adolescents who were born low birthweight to mothers with higher levels of education had lower executive function and higher behavioral problems scores compared with adolescents born low birthweight to women with lower levels of education (Table VI). Child sex did not modify the associations between perinatal exposures and adolescent development (data not shown).

## Discussion

We used data from a longitudinal follow-up study of a birth cohort in Tanzania to investigate the long-term relationships between perinatal outcomes and domains of adolescent neurodevelopment at 11-15 years of age. The results of this study suggest that gestational duration, birthweight, and birthweight for gestational age have a robust positive linear relationship with adolescent intelligence scores. Increased birthweight and birthweight for gestational age were also associated with higher executive function scores during adolescence; however, the association with behavioral problems score was more complex given an apparent U-shaped relationship. Compared with their normal birthweight counterparts, adolescents who were born low birthweight had lower intelligence and executive function scores and higher behavioral problems scores. Furthermore, the magnitude of associations between higher birthweight adjusted for gestational age with intelligence and executive function scores were significantly modified by maternal education level, such that adolescents born to women with higher levels of education had higher scores.

The positive association between continuous gestational age, birthweight, and birthweight for gestational age and intelligence score observed in this study are consistent with evidence from previous studies assessing the neurodevelopmental consequences of being born too soon or too small.^9,15,26^ For example, in a birth cohort of children born at full-term in Belarus, Yang et al observed a positive relationship between each week of gestational age and birthweight for gestational age and full-scale intelligence quotient at 6.5 years.^27^ Similarly, in a birth cohort of 505 healthy term-born children in South India, higher birthweight was also positively associated with higher child cognitive performance at 9-10 years of age.^28^ In Nepal, being born low birthweight or small for gestational age, but not preterm, was associated with deficits in general cognitive abilities and executive function in a birth cohort of 1923 children at 7-9 years of age, although this study did not examine the continuous relationships between gestational age and birthweight.^7^ Unlike the Nepal study, we did not observe an association between small for gestational age and adolescent neurodevelopment domains. This difference may be due to the much lower prevalence of small for gestational age in this study relative to the Nepal study (17% vs 55%, respectively), the substantially higher levels of maternal education in our study sample (>90% with ≥5-7 years of education vs a 21% literacy rate in the Nepal study), or the older age of adolescents in this study. Notably, in a recent study of 900 infants born in Sao Paolo, Brazil, there was no observed association between small for gestational age and neurodevelopment at 1 year of age, although preterm birth was significantly associated with poor neurodevelopmental scores.^29^ Therefore, although current evidence regarding the relationship between preterm birth and small for gestational age with neurodevelopmental scores later in life is mixed, a positive association between higher birthweight and cognitive development and executive function has been observed in several settings. In addition, higher birthweight for gestational age has been previously shown to be associated with lower risk of behavioral problems and improved prosocial behavior at 6.5 years of age in this cohort.^30^ The curvilinear associations between birthweight and birthweight for gestational age with behavioral problems score observed in this study, however, suggests that very low or very high birthweight may be associated with behavior problems in adolescence. However, given that measures of association between higher quartiles of birthweight and higher behavioral problems score crossed the null, it is possible that the true relationship may be a J-shaped, such that children born at low birthweight may have a higher behavioral problem score, with the relationship plateauing after normal birthweight threshold. Further evidence is therefore needed to clarify this relationship.

Nutritional insufficiency in utero is the leading biological mechanism explaining the link between birthweight, a proxy for fetal growth, and suboptimal neurodevelopment later in life.^31^ The “first 1000 days”—the duration of pregnancy and the first 2 years of life—are a sensitive period of rapid brain development.^32^ Data from animal and some human studies suggest that malnutrition in utero adversely affects neurodevelopmental processes, including neuron proliferation, axonal and dendritic growth, synaptogenesis, and myelination, as well as brain volume, leading to deficits in memory, learning, and higher order cognitive function.^31^ In line with this hypothesis, in a twin sibling study in Chile, birthweight was observed to be more strongly associated with fourth grade math and Spanish test scores (proxy for cognitive development) among monozygotic twins compared with dizygotic twins, suggesting that although genetic factors provide an important explanation for this difference, the competition for nutritional resources in utero may be less intense for dizygotic twins than monozygotic twins.^33^

A growing body of evidence also suggests that parental resources, particularly parental education, is protective against poor child development.^8^ In the twin study in Chile, higher maternal education attenuated the effect of birthweight on cognitive ability.^33^ The authors speculate that, in high-resource families, parental behavior may compensate for early biological disadvantage on educational achievement; whereas in low-resource families, parental behavior may reinforce early disadvantage by allocating more resources to a higher weight infant. Similarly, findings a study in rural India found that higher maternal resources, as measured by maternal literacy, and nurturance attenuated the association between poor linear growth and fine motor and receptive language development among preschool-aged children (<49 months), suggesting that maternal resources are protective again adverse nutritional exposures.^34^ These findings, however, are in contrast with observations from our study. The association between birthweight and adolescent neurodevelopmental scores in this study was attenuated among adolescents born to women with no education, suggesting that, in the context of poverty and low socioeconomic status overall, the relative contribution of birthweight to adolescent neurodevelopment is lower, whereas in an environment with higher maternal resources, as reflected by higher maternal education, the relative contribution of biological risks associated with birthweight for adolescent neurodevelopment become more apparent. In addition, it is also possible that the causes of low birthweight among women with higher levels of education may be more severe and differentially associated with neurodevelopmental outcomes compared with causes of low birthweight among women with lower levels ofeducation. As a result, low birthweight infants born to women with higher education are likely to have lower neurodevelopmental scores if the causes of low birthweight among women with high vs low education are more severe and more strongly related to poor developmental outcomes. In line with this hypothesis, low birthweight adolescents in this study had lower executive function scores and higher behavioral problems score, particularly among mothers with higher education. This association nonetheless is based on sparse data (ie, 16 low birthweight adolescents) and requires further data to confirm. Notably, the proportion of women with less than or equal to a primary school education level was substantially higher in this study (71%) compared with studies from Chile and India (approximately 25% in both), suggesting that the population in this study was generally of lower socioeconomic status. Therefore, maternal resources, as assessed by the proxy of education level, and environmental factors may differ substantially in this context than in previous studies. Further research is therefore needed to better understand how biological, nutritional, environmental, and parental caregiving practices may interact in different contexts to promote adolescent development.

Although a few studies have examined the shape of the association between gestational duration, birth weight, and child development, this study examined the shape of the relationship across the gradient of gestational duration and birthweight with adolescent neurodevelopment in sub-Saharan Africa.^30,35^ In addition, we evaluated multiple domains of adolescent neurodevelopment, including executive function and behavioral problems, for which evidence from low-income countries is sparse. However, the findings of this study should be interpreted in the context of its limitations. First, we cannot exclude the possibility of selection bias in this study given the lower survival probability among infants with adverse perinatal outcomes and the high loss to follow-up rate. Although, our primary inferences remained unchanged in sensitivity analyses using inverse probability weights to account for the loss to follow-up, we cannot rule out the possibility of selection bias. Therefore, evidence from population-based birth cohorts linking birth outcomes to adolescent development from the sub-Saharan African context are needed to confirm our findings. Second, although we used the East Africa Development tool, studies investigating the relationship between birth outcomes using other developmental assessment tools may lead to differing results owing to the heterogeneity between tools in scope and domains assessed.^36^ Third, gestational age at birth was assessed based on first day of last menstrual period as we did not have precise ultrasound-based assessment of gestational age; access to ultrasound examination at antenatal care is still rare in many LMIC settings. Nondifferential misclassification in gestational age, therefore, may have led to an attenuation of associations.^37^ Fourth, we did not have data on all factors which may influence birth outcomes and adolescent neurodevelopment (eg, prenatal maternal mental health); as such, we cannot rule out the risk of residual confounding associated with observational analyses. However, we adjusted for important sociodemographic confounders of the association between birth outcomes and adolescent development and did not adjust for any measures on the causal pathway to minimize the risk of bias. Finally, we did not have a direct measure of the quality of early learning opportunities in the home to be able to investigate the role of environment and caregiving in the association between birth outcomes and adolescent development.^34^

In summary, we observed that greater gestational duration and size at birth were associated with higher intelligence and executive function scores among Tanzanian adolescents at 11-15 years of age. Higher maternal education strengthened these associations, suggesting that in the context of overall low socioeconomic status, the relationship with birth size and neurodevelopment later in life are attenuated. The relationship between birthweight and adolescent socioemotional development, as captured by the behavioral problems score, was more complex and requires further investigation. Findings from this study nonetheless point to the importance of prenatal and postnatal interventions to prevent and support children who are born too soon or too small for optimal child and adolescent neurodevelopment. Prenatal maternal interventions that aim to improve birth outcomes may potentially mitigate the effect of biological insults in early life on neurodevelopment.^38^ In addition, increasing availability, access, and affordability of educational resources for young people who later become parents such that they are equipped with parental resources to support child development are crucial. However, to develop appropriate interventions and to scale-up programs to support early child development among infants with poor birth outcomes in the context of LMICs, further evidence from longitudinal studies is needed to understand the long-term impact of postnatal nutrition and child development interventions on human capital outcomes in later life.^39^ ■

## Figures and Tables

**Table I.** Maternal characteristics at trial enrollment and adolescent characteristics at birth and at the time of developmental assessment

Characteristic	Overall (n = 421)
Maternal age, years ± SD	28.1 ± 4.9
<20	21 (5.0)
20-24	95 (23)
25-29	174 (41)
≥30	131 (31)
Education, years	7.6 ± 2.9
0-4	34 (8.1)
5-7	266 (63)
8-11	93 (22)
≥12	28 (6.7)
Married or living with partner	386 (92)
Parity, no. of prior pregnancies	
None	54 (13)
1	157 (37)
2	103 (25)
≥3	107 (25)
Randomized supplementation group	
Placebo	223 (53)
Maternal multiple micronutrient	198 (47)
Maternal Hb at enrollment, g/dL^*^	
<8.5	45 (11)
8.5-10.9	182 (43)
≥11	120 (29)
Maternal BMI, kg/m^2†^	
<22	87 (21)
22-24.9	112 (27)
25-29.9	116 (28)
≥30	47 (11)
Maternal smoking, yes	3 (0.71)
Maternal alcohol consumption	
Never	336 (80)
Less than once per week	57 (14)
Once or more times per week	26 (6.2)
Antimalarial use (chloroquine), yes	16 (3.80)
Adolescent characteristics	
Female	213 (51)
Age at time of development assessment, years ± SD	13.1 ± 0.90
Preterm-born	55 (13)
Low birthweight	16 (3.80)
Small for gestational age	73 (17)
Birthweight and gestational age categories	
Term, average for gestational age	263 (63)
Term, small for gestational age	69 (16)
Term, large for gestational age	32 (7.6)
Preterm, average for gestational age	24 (5.7)
Preterm, small for gestational age	4 (0.95)
Preterm, large for gestational age	27 (6.4)

Values are number (%), unless otherwise noted.

* Missing data for 74 participants for baseline hemoglobin status.

† Body mass index (BMI) at enrollment was missing for 59 participants overall (5 missing for preterm-born children, 4 missing for low birthweight, and 15 missing for small for gestational age).

**Table II.** Baseline characteristics of women enrolled in the maternal multivitamin supplementation trial and whose children were enrolled and not enrolled in the follow-up study

Characteristics	Included in follow-up study (n = 421)	Enrolled in the trial, but lost to follow-up (n = 7919)	*P* value
Maternal age, years			
<20	21 (5.0)	1313 (17)	<.001
20-24	95 (23)	3226 (41)	
25-29	174 (41)	2080 (26)	
≥30	131 (31)	1295 (16)	
Maternal education, years			
0-4	34 (8.1)	917 (12)	.002
5-7	266 (63)	5286 (67)	
8-11	93 (22)	1311 (17)	
≥12	28 (6.7)	404 (5.1)	
Married or living with partner			
Parity, no. of prior pregnancies			
None	54 (13)	3707 (47)	<.001
1	157 (37)	2160 (27)	
2	103 (25)	1129 (14)	
≥3	107 (25)	915 (12)	
Randomized supplementation group			
Placebo	223 (53)	3964 (50)	.21
Maternal multiple micronutrient	198 (47)	3992 (50)	
Wealth quartile			
1st (lowest)	139 (33)	3176 (40)	<.001
2nd	53 (13)	1104 (14)	
3rd	118 (28)	2200 (28)	
4th (highest)	113 (27)	1451 (18)	
Maternal smoking, yes	3 (0.71)	22 (0.28)	.214
Maternal alcohol consumption			
Never	336 (80)	6953 (88)	<.001
Less than once per week	57 (14)	731 (9.2)	
Once or more times per week	26 (6.2)	233 (2.9)	
Child characteristics at birth			
Female	213 (51)	4043 (52)	.979
Preterm-born	55 (13)	1491 (19)	.004
Low birthweight	16 (3.80)	579 (7.9)	.001

**Table III.** Associations between birth outcomes and neurodevelopment scores among adolescents aged 11-15 years of age

	Intelligence score (n = 421)				Executive function score (n = 420)				Behavioral problems score (n = 420)			
Characteristics	Mean difference* (95% CI)	*P* value	Adjusted mean difference^†^ (95% CI)	*P* value	Mean difference* (95% CI)	*P* value	Adjusted mean difference^†^ (95% CI)	*P* value	Mean difference* (95% CI)	*P* value	Adjusted mean difference^†^(95% CI)	*P* value
Gestational age, weeks	0.05 (0.01 to 0.09)	.01	0.05 (0.01 to 0.09)	.02	0.03 (−0.004 to 0.07)	.08	0.03 (−0.01 to 0.07)	.18	−0.02 (−0.06 to 0.02)	.43	−0.02 (−0.06 to 0.02)	.29
Preterm birth												
No	Ref.		Ref.		Ref.		Ref.		Ref.		Ref.	
Yes	−0.28 (−0.56 to 0.00)	.05	−0.27 (−0.52 to 0.06)	.13	−0.26 (−0.54 to 0.03)	.08	−0.18 (−0.47 to 0.10)	.21	0.27 (−0.01 to 0.55)	.06	0.28 (−0.01 to 0.58)	.06
Birthweight,^‡^ per 100 g	0.04 (0.02 to 0.06)	<.001	0.04 (0.02.0.06)	<.001	0.03 (0.01 to 0.05)	.001	0.03 (0.01 to 0.05)	.001	–		–	
Birthweight quartile^‡^												
1st (≥3501 g)	–		–		–		–		0.15 (−0.13 to 0.43)	.29	0.12 (−0.16 to 0.41)	.39
2nd (3201-3500 g)	–		–		–		–		0.17 (−0.10 to 0.43)	.23	0.20 (−0.07 to 0.47)	.15
3rd (2900-3200 g)	–		–		–		–		Ref.		Ref.	
4th (≤2900 g)	–		–		–		–		0.26 (−0.01 to 0.52)	.06	0.30 (0.03 to 0.57)	.03
Low birthweight^‡^												
No	Ref.		Ref.		Ref.		Ref.		Ref.		Ref.	
Yes	−0.69 (−1.18 to −0.19)	.007	−0.63 (−1.14 to −0.11)	.02	−0.64 (−1.14 to −0.15)	.01	−0.58 (−1.09 to −0.08)	.02	0.72 (0.22 to 1.22)	.01	0.75 (0.24 to 1.27)	.004
Weight for gestational age,^‡^ SD	0.09 (0.005 to 0.17)	.04	0.09 (0.01 to 0.17)	.03	0.06 (−0.02 to 0.14)	.13	0.08 (−0.00 to 0.16)	.05	–		–	
1st (≥0.826)	–		–		–		–		0.17 (−0.11 to 0.44)	.23	0.16 (−0.12 to 0.44)	.27
2nd (0.825 to −0.073)	–		–		–		–		Ref.		Ref.	
3rd (−0.074 to −0.83)	–		–		–		–		0.16 (−0.11 to 0.44)	.27	0.19 (−0.09 to 0.47)	.18
4th (≤−0.83)	–		–		–		–		0.24 (−0.04 to 0.52)	.09	0.21 (−0.07 to 0.50)	.14
Small for gestational age^‡^												
No	Ref.		Ref.		Ref.		Ref.		Ref.		Ref.	
Yes	−0.07 (−0.33 to 0.19)	.61	−0.08 (−0.34 to 0.19)	.58	0.08 (−0.18 to 0.34)	.55	0.04 (−0.22 to 0.31)	.74	0.17 (−0.09 to 0.43)	.21	0.16 (−0.11 to 0.43)	.24

* Minimally aSMD in neurodevelopment scores during adolescence are adjusted for adolescent age (years) and sex (male vs female).

† Multivariable adjusted aSMD, adjusted for adolescent age (years), sex (male vs female), maternal age(<20, 20-24, 25-29, >30 years), maternal education (<5, 5-7, 8-11, ≥12 years), maternal marital status (married vs single/widowed/separated), maternal parity (none, 1 prior pregnancy, 2 prior pregnancies, ≥3 prior pregnancies), wealth quartile, alcohol consumption (never, less than once a week, once or more per week), and supplementation regimen (placebo vs micronutrient supplementation).

‡ Birthweight was missing for 2 infants in all analyses. Birthweight quartiles were used to estimate nonlinear associations as indicated by spline analyses.

**Table IV.** Associations between birth outcomes and neurodevelopment scores among adolescents aged 11-15 years of age

	Intelligence score (n = 421)				Executive function score (n = 420)				Behavioral problems score (n = 420)			
Characteristics	Mean difference* (95% CI)	*P* value	Adjusted mean difference^†^ (95% CI)	*P* value	Mean difference* (95% CI)	*P* value	Adjusted mean difference^†^ (95% CI)	*P* value	Mean difference* (95% CI)	*P* value	Adjusted mean difference^†^ (95% CI)	*P* value
Gestational age and size for gestational age^‡^												
Term-average for gestational age	Ref.		Ref.		Ref.		Ref.		Ref.		Ref.	
Preterm-average for gestational age	−0.45 (−0.87 to −0.04)	.03	−0.42 (−0.84 to 0.01)	.06	−0.29 (−0.71 to 0.13)	.18	−0.25 (−0.68 to 0.18)	.25	0.18 (−0.23 to 0.60)	.39	0.19 (−0.23 to 0.62)	.37
Term-small for gestational age	−0.06 (−0.33 to 0.22)	.67	−0.08 (−0.36 to 0.20)	.57	0.12 (−0.16 to 0.40)	.39	0.08 (−0.20 to 0.36)	.56	0.15 (−0.12 to 0.43)	.27	0.17 (−0.10 to 0.45)	.22
Preterm-small for gestational age	−0.77 (−1.76 to 0.22)	.13	−0.79 (−1.81 to 0.23)	.13	−1.0 (−2.0 to 0.01)	.05	−1.08 (−1.10 to −0.06)	.04	1.37 (0.38 to 2.36)	.01	1.38 (0.38 to 2.39)	.01

* Minimally aSMD in neurodevelopment scores during adolescence is adjusted for adolescent age (years) and sex (male vs female).

† Covariate aSMD adjusted for adolescent age (years), sex (male vs female), maternal age (<20, 20-24, 25-29, >30 years), maternal education (<5, 5-7, 8-11, >12 years), maternal marital status (married vs single/widowed/separated), maternal parity (none, 1 prior pregnancy, 2 prior pregnancies, 3 or more prior pregnancies), wealth quartile, alcohol consumption (never, less than once a week, once or more per week), and supplementation regimen (placebo vs micronutrient supplementation).

‡ Birthweight was missing for 2 infants in all analyses.

**Table V.** Associations between birth outcomes and neurodevelopment scores among adolescents aged 11-15 years of age accounting for censoring using inverse probability weights*

	Intelligence score (n = 421)		Executive function score (n = 420)		Behavioral problems score (n = 420)	
Characteristics	Adjusted mean difference^†^ (95% CI)	*P* value	Adjusted mean difference^†^ (95% CI)	*P* value	Adjusted mean difference^†^ (95% CI)	*P* value
Gestational age, weeks	0.06 (0.01 to 0.11)	.02	0.04 (−0.01 to 0.08)	.12	−0.001 (−0.05 to 0.05)	.97
Preterm birth	−0.35 (−0.71 to 0.01)	.06	−0.37 (−0.75 to 0.01)	.05	0.10 (−0.24 to 0.44)	.56
Birthweight,^‡^ per 100 g	0.06 (0.03 to 0.08)	<.001	0.05 (0.02 to 0.07)	<.001	–	
Birthweight quartile^‡^						
1st (≥3501 g)	–		–		0.32 (−0.01 to 0.65)	.06
2nd (3201 to 3500 g)	–		–		0.41 (0.05 to 0.77)	.03
3rd (2900 to 3200 g)	–		–		Ref.	
4th (≤2900 g)	–		–		0.62 (0.19 to 1.05)	.004
Low birthweight^‡^	−0.60 (−0.88 to −0.32)	<.001	−0.87 (−1.51 to −0.22)	.01	0.26 (−0.49 to 1.00)	.50
Weight for gestational age,^‡^ SD	0.10 (0.004 to 0.19)	.04	0.08 (−0.02 to 0.18)	.11	–	
1st (≥0.826)	–		–		0.04 (−0.28 to 0.37)	.79
2nd (0.825 to −0.073)	–		–		Ref.	
3rd (−0.074 to −0.83)	–		–		−0.18 (−0.56 to 0.20)	.35
4th (≤−0.83)	–		–		0.31 (−0.17 to 0.79)	.20
Small for gestational age^‡^	−0.17 (−0.49 to 0.15)	.29	0.06 (−0.29 to 0.42)	.72	0.12 (−0.35 to 0.59)	.62

* Adjusted for selection bias. Stabilized inverse probability of censoring weights (IPCW) were estimated as the ratio of the probability of being uncensored (ie, being included in the adolescent follow-up study) as a function of the adverse outcome (ie, exposure) over the probability of being uncensored conditional on the birth outcome and confounders (ie, adolescent age, sex, maternal age, maternal education, maternal marital status, maternal parity, wealth quartile, alcohol consumption, and supplementation regimen. Weights were derived for each domain of adolescent development and birth outcome separately.

† Marginal structural models with stabilized inverse probability censoring weights were used to estimate the adjusted mean difference in neurodevelopment scores as a function of birth outcomes.

‡ Birthweight was missing for 2 infants in all analyses.

**Table VI.** Predicted difference in adolescent neurodevelopmental domains as a function of birth outcomes by maternal education level

	Intelligence score (n = 421)		Executive function score (n = 420)		Behavioral problems score (n = 420)	
Characteristics	Adjusted mean difference* (95% CI)	*P* value	Adjusted mean difference* (95% CI)	*P* value	Adjusted mean difference* (95% CI)	*P* value
Among women with primary school education (7 years)^†^						
Preterm birth	−0.22 (−0.51 to 0.07)	.14	−0.20 (−0.48 to 0.09)	.18	0.34 (0.04 to 0.63)	.03
Low birthweight^‡^	−0.61 (−1.12 to −0.11)	.02	−0.58 (−1.08 to −0.08)	.02	0.77 (0.26 to 1.28)	.003
Small for gestational age^‡^	−0.05 (−0.32 to 0.21)	.71	0.07 (−0.19 to 0.33)	.60	0.15 (−0.12 to 0.34)	.27
Among women with secondary school education (12 years)^†^						
Preterm birth	−0.11 (−0.67 to 0.44)	.69	−0.26 (−0.81 to 0.29)	.36	0.70 (0.13 to 1.25)	.02
Low birthweight	−1.16 (−1.97 to −0.36)	.01	−1.28 (−2.08 to −0.49)	.002	1.58 (0.76 to 2.39)	<.001
Small for gestational age	−0.40 (−0.95 to 0.14)	.15	−0.39 (−0.93 to 0.15)	.16	−0.02 (−0.57 to 0.54)	.96
*P* value for interaction between maternal education and preterm birth^§^	0.67		0.79		0.12	
*P* value for interaction between maternal education and low birthweight^§^	0.10		0.03		0.02	
*P* value for interaction between maternal education and small for gestational age^§^	0.15		0.06		0.51	

* Multivariable aSMD, adjusted for adolescent age (years), sex (male vs female), maternal age (<20, 20-24, 25-29, >30 years), maternal marital status (married vs single/widowed/separated), maternal parity (none, 1 prior pregnancy, 2 prior pregnancies, 3 or more prior pregnancies), wealth quartile, alcohol consumption (never, less than once a week, once or more per week), and supplementation regimen (placebo vs micronutrient supplementation).

† We estimated the SMD with 95% CI for association between of adverse birth outcomes and development scores among adolescents born to women with 7 years education compared to 12 years of education, by estimating the marginal effect at 5 years and 12 years education based on pooled models with linear interaction terms between perinatal outcomes and maternal education as a continuous term.

‡ Birthweight was missing for 2 infants in all analyses.

§ Wald-test *P* values are based on pooled models with linear interactions terms with maternal education.

## Abbreviations

aSMD: adjusted standardized mean difference
BRIEF: Behavior Rating Inventory of Executive Function
LMIC: Low- and middle-income country
SDQ: Strengths and Difficulties Questionnaire
SMD: Standardized mean difference
